# Central blockade of NLRP3 reduces blood pressure via regulating inflammation microenvironment and neurohormonal excitation in salt-induced prehypertensive rats

**DOI:** 10.1186/s12974-018-1131-7

**Published:** 2018-03-24

**Authors:** Mo-Lin Wang, Yu-Ming Kang, Xiao-Guang Li, Qing Su, Hong-Bao Li, Kai-Li Liu, Li-Yan Fu, Roland Osei Saahene, Ying Li, Hong Tan, Xiao-Jing Yu

**Affiliations:** 1 Department of Physiology and Pathophysiology, Xi’an Jiaotong University School of Basic Medical Sciences, Key Laboratory of Environment and Genes Related to Diseases (Xi’an Jiaotong University), Ministry of Education, Xi’an, 710061 China; 2 Department of Immunology, School of Basic Medical Sciences, Jiamusi University, Jiamusi, 154007 China; 3 Department of Rehabilitation Medicine, People’s Hospital of Baoan District, Shenzhen, 518100 China; 4 Department of Pathology, Xi’an Jiaotong University School of Basic Medical Sciences, Xi’an Jiaotong University Health Science Center, Xi’an, 710061 China

**Keywords:** NLRP3, Hypothalamic paraventricular nucleus, Inflammation, Neurotransmitters, Microglia, Hypertension

## Abstract

**Background:**

Inflammation has been implicated in the development of cardiovascular disease. We determined whether nod-like receptor with pyrin domain containing 3 (NLRP3) involved in the process of prehypertension, central blockade of NLRP3 decreased inflammation reaction, regulated neurohormonal excitation, and delayed the progression of prehypertension.

**Methods:**

Prehypertensive rats were induced by 8% salt diet. The rats on high-salt diet for 1 month were administered a specific NLRP3 blocker in the hypothalamic paraventricular nucleus (PVN) for 4 weeks. ELISA, western blotting, immunohistochemistry, and flow cytometry were used to measure NLRP3 cascade proteins, pro-inflammation cytokines (PICs), chemokine ligand 2 (CCL2), C-X-C chemokine receptor type 3 (CXCR3), vascular cell adhesion molecule 1 (VCAM-1), neurotransmitters, and leukocytes count detection, respectively.

**Results:**

NLRP3 expression in PVN was increased significantly in prehypertensive rats, accompanied by increased number of microglia, CD4^+^, CD8^+^ T cell, and CD8^+^ microglia. Expressions of PICs, CCL2, CXCR3, and VCAM-1 significantly increased. The balance between 67-kDa isoform of glutamate decarboxylase (GAD67) and tyrosine hydroxylase (TH) was damaged. Plasma norepinephrine (NE) in prehypertensive rats was increased and gamma-aminobutyric acid (GABA) was reduced. NLRP3 blockade significantly decreased blood pressure, reduced PICs, CCL2, VCAM-1 expression in PVN, and restored neurotransmitters. Blood pressure and inflammatory markers were upregulated after termination of central blockage NLRP3.

**Conclusions:**

Salt-induced prehypertension is partly due to the role of NLRP3 in PVN. Blockade of brain NLRP3 attenuates prehypertensive response, possibly via downregulating the cascade reaction triggered by inflammation and restoring the balance of neurotransmitters.

**Electronic supplementary material:**

The online version of this article (10.1186/s12974-018-1131-7) contains supplementary material, which is available to authorized users.

## Background

Recent evidences have associated hypertension with a chronic low-grade systemic inflammation [1].The most susceptible among all organs to increased blood pressure is the brain [2]. The hypothalamic paraventricular nucleus (PVN) plays a key role in endocrine-autonomic control by regulating baroreflex function, sympathetic output, and salt appetite [3]. Furthermore, growing body of evidence have demonstrated that increased pro-inflammatory cytokines (PICs) within PVN play an important role in the progression of hypertension [4, 5]. Researches described that hypertension is associated with increased Toll-like receptor 4 (TLR4) expression in the PVN of essential and angiotensin II induced hypertensive rats [6, 7]. However, the role of intracellular NOD like receptors, such as pyrin domain containing 3 (NLRP3) in hypertension remains unknown. Then, in this study, we analyze the role of NLRP3, PICs, chemokine ligand 2 (CCL2), C-X-C chemokine receptor type 3 (CXCR3), vascular cell adhesion molecule 1 (VCAM-1), and neurohormone level in the development of prehypertension.

Upon activation, NLRP3 inflammasome is formed when NLRP3 recruits the adapter protein apoptosis-associated speck-like protein (ASC) and pro-cysteine aspartic acid protease-1 (pro-caspase-1). The NLRP3 inflammasome is assembled and matured under different exogenous and endogenous activators, resulting in the production of interleukin—1 beta (IL-1β) and interleukin—18 (IL-18) [8]. IL-1β is a multifunctional cytokine which contributes to chronic inflammation and induces strong inflammatory response during cardiovascular diseases [9–12]. Several physiological and endocrine adjustments induced by immune activation are mediated and performed by IL-1β in the brain [13]. Recent study also suggests that blood pressure and serum norepinephrine level is increased when IL-1β was injected into the brain [14].

The state of inflammation and immune activation perpetuated in hypertension may affect severity and progression of tissue and organ injury. An excessive inflammatory response is well-recognized in determining hypertension process and hypertensive brain damage [15]. Activation of monocytes correlates with hypertension in the periphery and increased plasma pro-inflammatory cytokines [16–18]. The activation of brain-resident astrocytes, microglia, and upregulation of adhesion molecules expression on brain endothelial cells occur as a result of increased blood pressure [19, 20]. The expression of ICAM-1 and VCAM-1 by endothelial cells further promote inflammation [21]. CCL2 is also called monocyte-chemotactic protein (MCP-1), which was upregulated in the kidneys of deoxycorticosterone acetate (DOCA) salt-hypertensive animals [22]. Recent studies have shown that chemokine CXCR3 and its corresponding chemokine ligands, CXCL9, CXCL10, and CXCL11, are expressed in central nervous system diseases [23–25]. CXCR3 is a well-known tissue-homing chemokine for T cells, which is highly expressed in the circulation of hypertensive patients. CD4^+^ and CD8^+^ T cells in hypertensive patients have increased renal infiltration than control group [26].

Accordingly, we hypothesized that NLRP3 activation was associated with hypertension, upregulation of pro-inflammatory cytokines IL-1β, interleukin-6 (IL-6), tumor necrosis factor-α (TNF-α); adhesion molecule VCAM-1, chemokine CCL2, CXCR3 in PVN, and microglial activation, contribute to the inflammatory reaction with amplification, which resulted in an imbalance of excitatory and inhibitory neurotransmitters, increase of sympathetic excitability and elevated blood pressure. Furthermore, we postulated that blockade of NLRP3 in PVN could have important functional consequences and lead to an associated decrease in hypertension by regulating inflammation microenvironment and neurotransmitters. This research will reveal a potential new therapeutic target for the treatment of hypertension.

## Methods

### Experimental animals

The experimental animals were made up of male Sprague-Dawley (SD) rats (250–270 g). The rats were kept and maintained at temperatures of (20–23 °C) under controlled 12 h/12 h dark/light cycle. The experimental rules and regulations in accordance with the National Institutes of Health Guide for the Care and Use of Laboratory Animals were duly followed. The approval for our study was obtained from the Xi’an Jiaotong University Committee for Animal Research.

### General experimental protocol

0.3% NaCl and 8% NaCl were administered to normal-salt (NS) group and the high-salt (HS) group over a period of 3 months, respectively. The rats were divided into 10 groups: (i) normal salt 2 months (NS2), (ii) high salt 2 months (HS2), (iii) normal salt 3 months (NS3), (iv) high salt 3 months (HS3), (v) NS + Vehicle, (vi) NS + MCC950, (vii) HS + Vehicle, (viii) HS + MCC950, (ix) NS + MCC950’, (x) HS + MCC950’.

After high-salt diet for 4 weeks, bilateral cannulae were implanted into the PVN of (v), (vi), (vii), (viii), (ix), and (x) groups rats for infusion of MCC950 (15 μg/h, Medchem Express), a specific NLRP3 blocker, or vehicle (artificial cerebrospinal fluid, aCSF). The dose applied for MCC950 was assessed from a study in rats with doses of 3, 15, and 65 μg/h [27, 28]. The highest dose caused mortality while the 15 μg/h produced optimal response but the low dose recorded an incomplete inhibition. After 4 weeks of drug intervention, at the end of 8 weeks rats of (v), (vi), (vii), and (viii) groups were administered an anesthesia of ketamine (80 mg/kg) and xylazine (10 mg/kg) mixture (ip); following this, the brains, hearts, aortas, and peripheral blood were removed and immediately frozen on dry ice. But (ix) and (x) groups were kept, and the rats were fed with high-salt diet for another 1 month without treatment until the end of the experiment.

### The intra-paraventricular nucleus cannula application for chronic infusion

A 28-day mini osmotic pump (infusion rate 0.25 μl/h; Alzet, model 2004, Durect Corporation, Cupertino, CA, USA) was through a catheter tube connected to the infusion cannula to deliver MCC950 or vehicle in the PVN, as described previously [29].

### Blood pressure measurements

The noninvasive computerized tail-cuff system (NIBP, AD Instruments, Australia) was applied for measuring blood pressure of the tail artery in conscious rats, as described previously in our report [30].The mean blood pressure of all rats were determined and recorded per week.

### Hematoxylin and eosin staining

5 μm pathology slices from the brain, aorta, and heart were prepared to discern morphology by staining with hematoxylin and eosin (H&E).

### Immunohistochemistry staining

The following antibodies were immunohistochemically determined: NLRP3 (1:200, Santa Cruz, CA, USA), IL-1β (1:200, Santa Cruz, CA, USA), ASC (1:100, Santa Cruz, CA, USA), pro-casp-1 (1:100, Santa Cruz, CA, USA), CD8^+^ (1:200, Santa Cruz, CA, USA), Iba-1 (1:200, Santa Cruz, CA, USA), TH (1:200, Santa Cruz, CA, USA), and GAD67 (1:200, Santa Cruz, CA, USA). The detailed immunostaining protocol performed was the same as in our previous research [29, 31]. The Image-Pro Plus software was applied in the analysis of the integral optical density and fluorescence intensity.

### Isolation of peripheral blood mononuclear cells (PBMCs)

4 ml of Ficoll-Hypaque were added to 50 U/ml heparinized venous bloods and centrifuged at 400 g for 30 min at room temperature. PBMC interface layer was washed in phosphate buffer saline (PBS) twice and was centrifuged for 10 min at 300 g. RPMI medium supplemented with fetal calf serum was used to resuspend cells to a final concentration of 1 × 10^6^ monocytes/ml [32].

### Western blotting

The brain was sectioned serially in 300 μm increments from the bregma to lambda, both sides of the PVN tissues were isolated by the use of a punch-out technique with a cryostat [33, 34], and the PVN tissue was stored at − 80 °C until use. Western blotting analysis was performed in the same manner as previously described [6]. The protein levels were determined from tissue homogenate obtained from the PVN for the following antibodies: NLRP3 (1:2000, Santa Cruz, CA, USA), ASC (1:500, Santa Cruz, CA, USA), pro-caspase-1 (1:2000, Abcam, MA, USA), IL-1β (1:500, Santa Cruz, CA, USA), CXCR3 (1:2000, Abcam, MA, USA), VCAM-1, ICAM-1 (1:2000, Abcam, MA, USA), and CCL2 (1:2000, Santa Cruz, CA, USA), Iba-1 (1:500, Santa Cruz, CA, USA). The β-actin antibody was used as an internal standard, and band densities were analyzed with NIH ImageJ software.

### Flow cytometric analysis of leukocyte in PBMCs

PBMCs were transferred to a tube and stained with an antibody cocktail consisting of CD3, CD4, and CD8 (BD Bioscience, USA) diluted in PBS. Samples were then analyzed by flow cytometry using a FACS Calibur TM cytometer (BD Biosciences, San Jose, CA, USA).

### Cytokines measure

Commercially available rat ELISA kits were used to quantify tissue TNF-α, IL-6, and plasma norepinephrine (Invitrogen Corporation, CA, USA) following the manufacturer’s instructional manual.

### Data analysis

The data were recorded as mean ± SEM. Data were analyzed between two groups using the Student’s *t* test. For experiments that involved multiple groups, data analyses were conducted by either a one-way or two-way ANOVA followed by a post hoc Bonferroni test. A probability value of *P* < 0.05 was inferred to be statistically significant.

## Results

### The role of NLRP3 on blood pressure in salt-induced prehypertensive rats

**Fig. 1:**
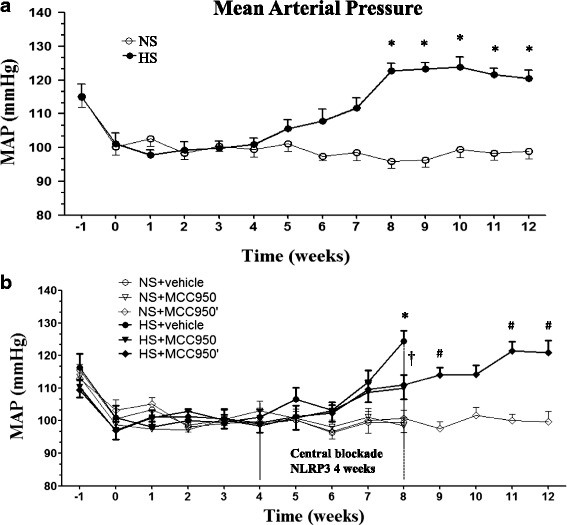
Effects of NLRP3 on MAP in salt-induced prehypertension rats. **a** The blood pressure of the high-salt group increased gradually after 2 months of high-salt diet in comparison with control group. **b** Compared with HS + vehicle rats, chronic PVN infusion of MCC950 a specificity NLRP3 antagonist for 4 weeks attenuated hypertension. Blood pressure continued to arose after 4 weeks of discontinuation treatment. Values are expressed as means ± SEM, *n* = 6–8. *P < 0.05, NS vs HS, or NS + vehicle vs HS + vehicle, †*P* < 0.05, HS + MCC950 vs HS + vehicle, #*P* < 0.05, HS + MCC950’ vs NS + MCC950’

The initial blood pressure baseline levels were similar. By the fourth week of high-salt diet, the blood pressure of high-salt diet rats had gradually increased. The MAP of high-salt group increased significantly after 8 weeks compared with NS groups (Fig. 1a). Chronic PVN infusion of MCC950, a specific NLRP3 antagonist for 4 weeks of high-salt diet, blood pressure of HS + MCC950 group was no longer increased; however, the MAP of HS + MCC950’ group was increased compared with the control group, after the central occlusion of NLRP3 was stopped and the high salt feeding for another 4 weeks (Fig. 1b).

### The expression of NLRP3/ASC/IL-1β in the PVN of high-salt diet rats by the second month

**Fig. 2:**
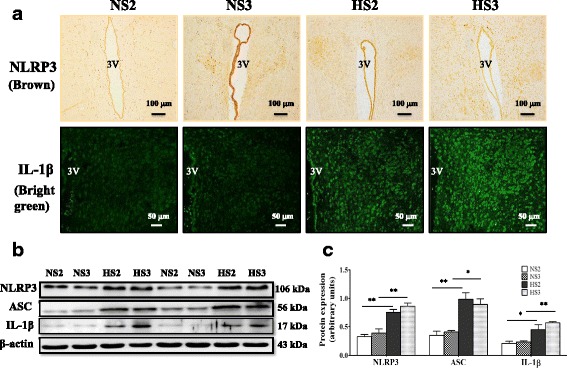
The expression of NLRP3 pathway protein in PVN. **a** A representative immunohistochemistry image of NLRP3, scale bar 100 μm, immunofluorescence image of IL-1β, scale bar 50 μm, showing the changes of NLRP3, IL-1β in the high-salt diet rats from 2 to 3 months. **b** A representative immunoblot of NLRP3/ASC/IL-1β. **c** Analysis of NLRP3 pathway expression in various groups; *n* = 3. Values are expressed as means ± SEM. *P < 0.05, **P < 0.001, vs control (NS), 3 V, third ventricle

In this study, we found that NLRP3, ASC, and IL-1β expressions in the PVN of high-salt diet increased significantly by the second month, with a corresponding increase in the blood pressure. Most interestingly, the NLRP3 pathway proteins expression had no difference between the high-salt diet for 2 and 3 months (Fig. 2).

### The expression of NLRP3 in the heart, aorta, and plasma pro-inflammatory cytokines in HS group by the second month

**Fig. 3:**
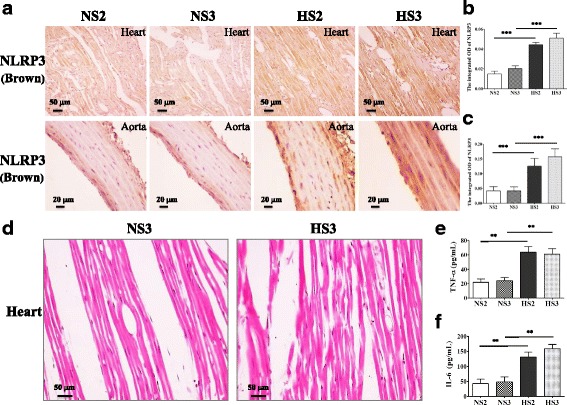
**a** A representative immunohistochemistry image of NLRP3 in the heart and aorta, scale bar 50 and 20 μm, showing the changes of NLRP3 in the high-salt diet rats from 2 to 3 months. **b** Analysis integrated OD of NLRP3 in various heart tissue groups, *n* = 6–8. **c** Analysis integrated OD of NLRP3 in various aorta tissue groups, *n* = 6–8. **d** The tissue sections of the left ventricles were stained by HE staining, scale bar 50 μm. **e** ELISA detection plasma TNF-α expression in the various groups, *n* = 6–8. **f** ELISA detection plasma IL-6 expression in the various groups, *n* = 6–8.Values are expressed as means ± SEM. **P < 0.001, ***P < 0.0001 vs control (NS)

Our study demonstrated that HS group had shown significantly increased NLRP3 in the heart and aorta since the second month, as shown in Fig. 3a–c.* ELISA* results showed that plasma TNF-α and IL-6 in the HS groups by the second month were higher than those in the NS rats (Fig. 3e, f).

Morphologically, we observed that the cardiac myocytes nucleus enlarged in the HS model since the third month, which indicated early myocardial hypertrophy (Fig. 3d).

### The number of microglia was increased in the cortex and hippocampus of rats with high salt for 2 and 3 months

**Fig. 4:**
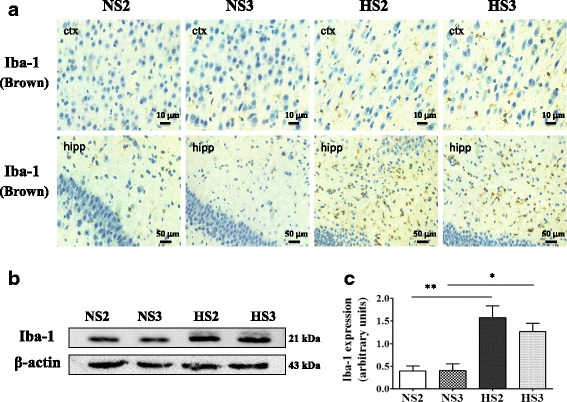
The expression of Iba-1 in the microglia was increased. **a** A representative immunohistochemistry image of Iba-1 in cortex and hippocampus, scale bar 10 and 50 μm. **b** A representative immunoblot of Iba-1. **c** Analysis of Iba-1 expression in various groups, *n* = 3. Values are expressed as means ± SEM. *P < 0.05, **P < 0.001, vs control (NS)

Immunohistochemistry and western blotting showed that the number of microglia in hippocampus and cortex of the HS group was higher than that of the control group (Fig. 4).

### CD4^+^ and CD8^+^ T cells in PBMCs and CD8^+^ microglias/macrophages in the brain were increased in of high-salt diet rats since the third month

**Fig. 5:**
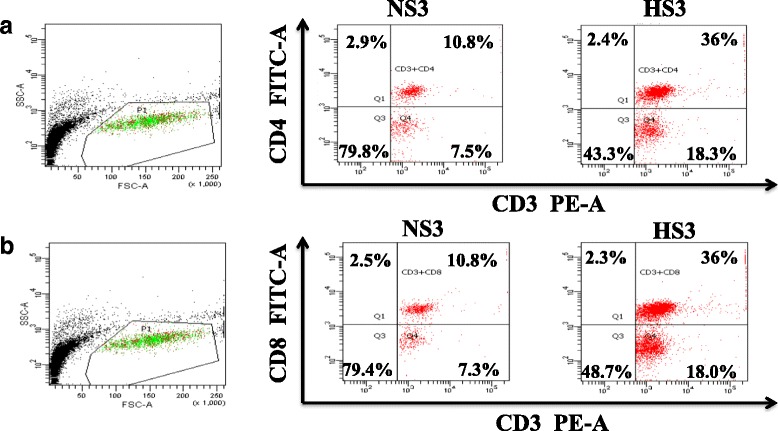
Flow cytometric analysis T cells in peripheral circulation. **a** CD4^+^ T cell (CD3^+^CD4^+^). **b** CD8^+^ T cell (CD3^+^CD8^+^). Data is represented as the percentage of CD3^+^CD4^+^, CD3^+^CD8^+^

**Fig. 6:**
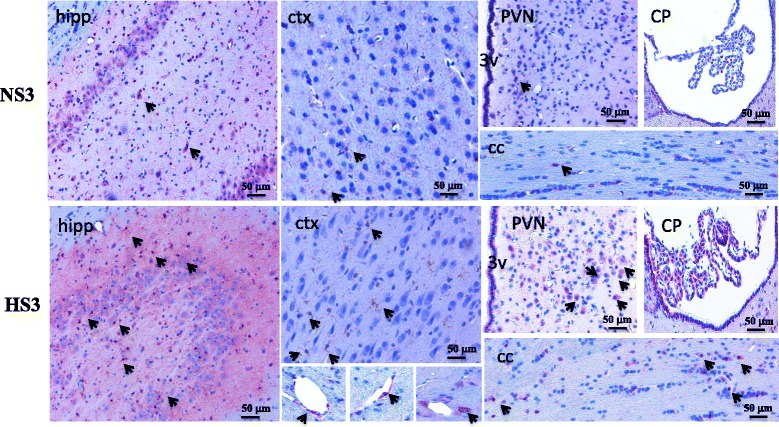
Increased appearing of CD8^+^ microglias/macrophages in the brain of high-salt diet rats. A representative immunohistochemistry image of CD8 molecule microglias/macrophages and neurons in cortex (ctx), corpus callosum (cc), hippocampus (hipp), choroid plexus (CP), and hypothalamic paraventricular nucleus (PVN), scale bar 50 μm, showing the changes of CD8^+^ cells in the high-salt diet rats for 3 months, *n* = 6–8, 3 V, third ventricle

We performed flow cytometric analysis to determine leukocyte numbers in PBMCs. Compared to NS rats, the number of T cell in PBMCs was elevated in HS rats. Further analysis of T cell subsets showed that the populations of CD4^+^ and CD8^+^ T cells increased significantly (Fig. 5). In peripheral circulation, the expression of CD4^+^ and CD8^+^ T cells increased, and expression of CXCR3 in PVN was upregulated. Therefore, we sought to find infiltrating T cells in the brain parenchyma. We did not find infiltrating T cells in the brain, but we found microglias/macrophages expressing CD8 molecules in the cortex, corpus callosum, hippocampus, choroid plexus, as well as neurons expressing CD8 molecules in hippocampus and PVN. Jander has previously reported the expression of CD8^+^ macrophages in the central nervous system [35, 36] (Fig. 6).

### Blockade NLRP3 suppression of ASC/pro-caspase-1/IL-1β expression in PVN of high-salt diet rats

**Fig. 7:**
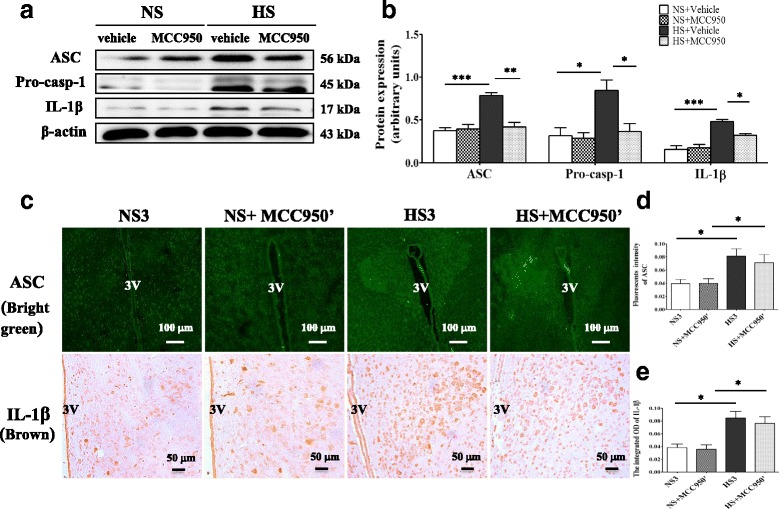
Central blockade NLRP3 decreases ASC/Pro-caps-1/IL-1β expression of prehypertensive rats. **a** A representative western blot of NLRP3 pathway. **b** Densitometric analysis expression of NLRP3 pathway in the PVN of different groups, *n* = 3. **c** A representative immunofluorescence image of ASC, scale bar 100 μm, and immunohistochemistry image of IL-1β, scale bar 50 μm, showing the changes of ASC and IL-1β expression when central blockade NLRP3 was stopped. **d** Densitometric analysis of immunofluorescent intensity of ASC in various groups. **e** Densitometric analysis of integrated OD of IL-1β in various groups, *n* = 6–8. Values are expressed as means ± SEM. *P < 0.05, **P < 0.001, ***P < 0.0001, 3 V, third ventricle

To determine whether suppression of NLRP3 in PVN attenuates inflammation response, we examined the protein level of NLRP3 pathway in PVN. Chronic PVN infusion of MCC950 significantly decreased ASC, pro-caspase-1, and IL-1β expression in high-salt diet rats (Fig. 7a, b). However, when the drug intervention was stopped and continued with high-salt feeding for a month, we observed that the ASC and IL-1β of HS + MCC950’ group was significantly increased in PVN than those of control group (Fig. 7c–e).

### Central blockade NLRP3 inhibited VCAM-1, CXCR3, CCL2, and PICs expression in the PVN of high-salt diet rats

**Fig. 8:**
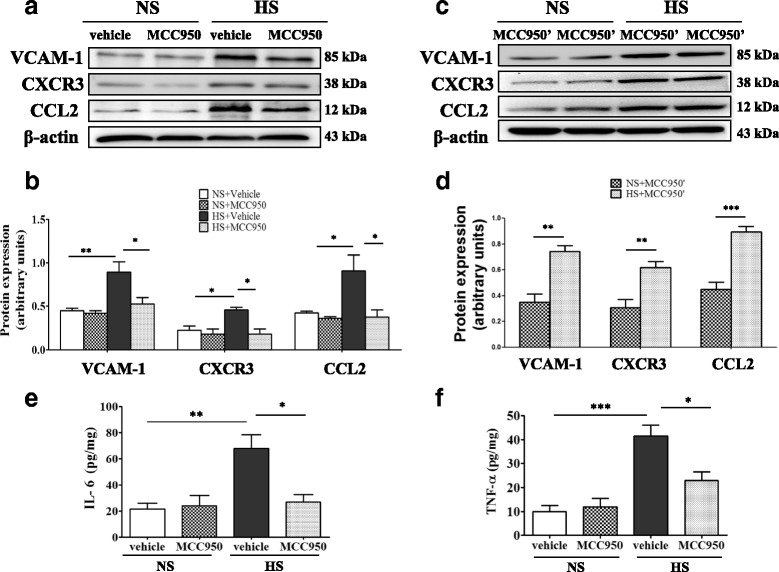
Central blockade NLRP3 suppresses of VCAM-1, CXCR3, and CCL2 expression in the PVN of prehypertensive rats. **a** A representative immunoblot of VCAM-1, CXCR3, and CCL2. **b** Densitometric expression analysis of VCAM-1, CXCR3, and CCL2 in various groups, *n* = 3. **c** Terminated treatment of anti-NLRP3, a representative immunoblot of VCAM-1, CXCR3, and CCL2. **d** Densitometric protein analysis of VCAM-1, CXCR3, and CCL2 in various groups; *n* = 3. **e** ELISA detection IL-6 expression in the PVN of the various groups, *n* = 6–8. **f** ELISA detection TNF-α expression in the PVN, *n* = 6–8. Values are expressed as means ± SEM. *P < 0.05, **P < 0.001. ***P < 0.0001

Western blotting revealed that several inflammatory markers adhesion molecule VCAM-1, chemokine CXCR3, and CCL2 were increased in prehypertensive rats compared to NS rats. Central blockade NLRP3 attenuated VCAM-1, CXCR3, and CCL2 expression (Fig. 8a, b). Termination of intervention and continued high-salt feeding for a month, led to significant upregulation VCAM-1,CXCR3, and CCL2 in the MCC950’ treatment group, as shown in Fig. 8c, d. ELISA analysis showed that HS + vehicle group had significant increase in IL-6 and TNF-α expression in the PVN compared with NS + vehicle group. The upregulation of TNF-α and IL-6 were significantly attenuated by central blockade NLRP3 (Fig. 8e, f).

### Central blockade NLRP3 regulates PVN excitatory and inhibitory neurotransmitters and plasma norepinephrine and GABA

**Fig. 9:**
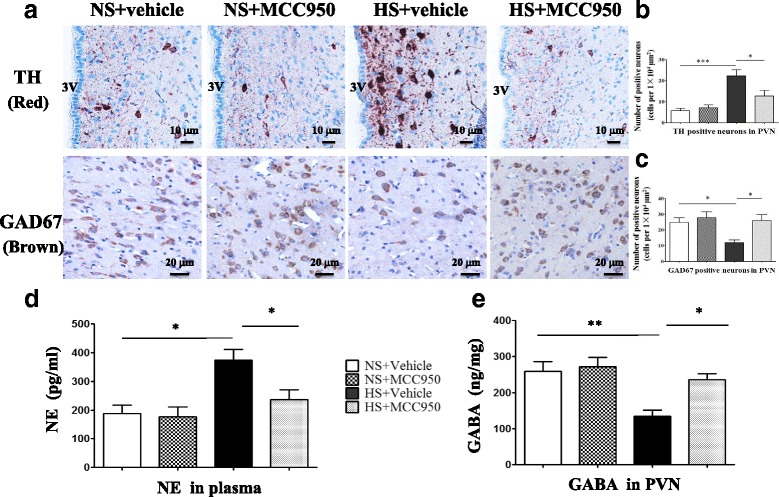
Central blockade NLRP3 regulates PVN excitatory and inhibitory neurotransmitters. **a** A representative immunohistochemistry image of TH and GAD67, scale bar 10 and 20 μm, showing the changes of TH and GAD67 in the high-salt diet rats after central blockade NLRP3. **b** Expression of TH in various groups; *n* = 6–8. **c** Expression of GAD67 in various groups, *n* = 6–8. **d** ELISA detection plasma NE expression in various groups, *n* = 6–8. **e** ELISA detection GABA expression in various groups, *n* = 6–8.Values are expressed as means ± SEM. *P < 0.05, ***P < 0.0001, 3 V, third ventricle

Compared with control groups, hypertensive rats had higher expression of TH and lower expression of GAD67 in the PVN. Chronic infusion of MCC950 into the PVN for 4 weeks decreased the expression of TH and increased GAD67 expression in prehypertensive rats induced by salt. Plasma NE in prehypertensive rats was increased and GABA was reduced; interestingly, central blockade of NLRP3 significantly decreased plasma NE and upregulated GABA levels in PVN (Fig. 9).

## Discussion

In this study, the role of NLRP3 expression in the PVN of salt-induced prehypertension was defined. The novel outcomes of this present research are the following: (1) NLRP3 expression in the PVN of high salt was increased with corresponding elevated blood pressure, (2) NLRP3 activation in the PVN was accompanied by high expression of PICs, adhesion molecule VCAM-1, chemokine CCL2 and CXCR3, as well as breaking the balances of neurotransmitters in the PVN, (3) microglial activation, CD8^+^ microglias/macrophages were increased in brain parenchyma of prehypertensive rats, (4) central blockade of NLRP3 in the PVN attenuated prehypertension resulting from regulating the inflammation microenvironment and restoring the balances of neurotransmitters in PVN, (5) NLRP3 was not only upregulated in the PVN, but also highly expressed in the peripheral tissue, heart and aorta.

PVN in the brain is notable as an important endocrine-autonomic control area which contributes to sympathetic regulation of blood pressure and body fluid homeostasis [37]. The sympathetic outflow from the PVN depends on the balance of different kinds of neurotransmitter activities. Increasingly evidences demonstrated that the increased sympathetic activation is due to an increase in excitatory adrenergic and glutamatergic activities and a decrease in GABAergic activity in the PVN [38]. In chronic sterile inflammation, inflammatory responses are caused and maintained by the NLRP3 inflammasome [39], which activation of pro-caspase-1 and IL-1β, IL-1β is recognized as the main activator of inflammation, which serves as a mediator to trigger the cascade release of other cytokines. Reports have demonstrated that various PICs such as TNF-α, IL-1β, and IL-6 play a vital role in the development of hypertension [31, 40–42]. Tissue cells synthesize inflammatory mediators and chemokine in response to injury and stress, to recruit inflammatory cells. Positive feedback loops result as inflammatory cells perpetuate the release of cytokines and express adhesion molecules. Studies have shown that adhesion molecules ICAM-1, VCAM-1, immune cells, chemokine CCL2, and CXCR3 are key factors in human hypertension [21, 22, 26]; these series of events results in the progression of hypertension.

In this study, we provide the evidence that NLRP3 plays a key role in prehypertension induced by salt. Our study demonstrated that HS rats recorded a significant increase in MAP in comparison with NS group by the 2 months of high-salt diet, and the changes were paralleled with NLRP3 increased expression in HS group. HS group feed with high salt for 2 months had significant increase in NLRP3 not only in the PVN but also in the heart and aorta peripheral tissues, as well as upregulated plasma IL-6 and TNF-α.We also found that VCAM-1, chemokine CCL2, and CXCR3 in the PVN were increased since the second month of high-salt diet. During inflammatory responses, chemokine classically mediate, migrate, and traffic cells to sites of inflammation [43]. Arterial hypertension affects bone marrow hematopoietic niche directly by increased levels of monocytes and neutrophils in the circulation [44]. In fact, previous studies showed that the blood and brain of naive SHR expressed increased monocyte and neutrophil counts [45–47]. Our data illustrated that the number of microglia in the cortex and hippocampus and total T cells in the peripheral circulation in HS rats were increased compared to NS rats, and further analysis of the immune cell subsets revealed that the populations of CD4^+^ and CD8^+^ T cells were increased significantly.

We try to find the infiltration of T cells in the brain parenchyma without success, but we found the number of CD8^+^ microglias/macrophages in the brain increased. Both CD4 and CD8 antigens were originally described as antigen coreceptors on helper and cytotoxic/suppressor T lymphocytes, respectively. However, Jander et al. described a population of activated central nervous system (CNS) macrophages characterized by the expression of the CD8 molecule [35, 36].

Our next step sought to determine whether the anti-NLRP3 therapy could decrease cascade reaction of inflammation, regulate neurotransmitters, and counteract hypertension. We found that blockade NLRP3 therapy markedly reversed hypertension, in consistent with the results reported by Krishnan [48], as well as significantly reduced NLRP3 pathway protein expression, decreased PICs, CCL2, CXCR3, and VCAM-1, adjusted TH, GAD67, and GABA in PVN and plasma NE. However, when we stopped the drug intervention and continued feeding with the high-salt diet for a month, we observed that HS + MCC950’ group had significant activation of ASC, IL-1β, increased VCAM-1, CCL2, and CXCR3 expression again in the PVN compared to the control group.

## Conclusions

In summary, our results show that NLRP3 plays a key role in prehypertensive rats induced by high-salt diet, via an inflammatory mechanism that increases IL-1β, IL-6, and TNF-α; chemokine CCL2, CXCR3; adhesion molecule VCAM-1 and adjusts excitatory neurotransmitters and inhibitory neurotransmitters, as shown in Additional file 1: Figure S1. These findings shed new lights into the molecular mechanism that controls hypertension; however, whether NLRP3 could be used as a therapeutic target for hypertension remains to be clarified.

## Limitations

Our study has limitations. We observed that adhesion molecule VCAM-1 and chemokine CCL2, CXCR3 are highly expressed in the PVN; however, it is unclear which cells express these molecules, endothelial cells, glial cells or infiltrating leukocytes? Then, what activates the NLRP3? What are the first steps that trigger the inflammation response in the PVN? All these questions needs to be further explored and discussed.

## Additional file

Additional file 1: **Figure S1.** Salt-induced prehypertension is partly due to the role of NLRP3 in PVN, via an inflammatory mechanism. Blockade of brain NLRP3 attenuates prehypertensive response, possibly via downregulating the cascade reaction triggered by inflammation and restoring the balance of neurotransmitters (PPTX 203 kb)

## Abbreviations

ASC: Adapter protein apoptosis-associated speck like protein
BP: Blood pressure
CCL2: Chemokine ligand 2
CXCR3: C-X-C chemokine receptor type 3
DAMP: Danger-associated molecular pattern
DOCA: Deoxycorticosterone acetate
GABA: Gamma-aminobutyric acid
GAD67: 67-kDa isoform of glutamate decarboxylase
Iba-1: Ionized calcium-binding adaptor molecule-1
ICAM-1: Intercellular adhesion molecule 1
IL-18: Interleukin 18
IL-1β: Interleukin 1β
IL-6: Interleukin 6
MAP: Mean arterial pressure
mmHg: Millimeter of mercury
NF-κB: Nuclear factor-kappa B
NLRP3: Nod-like receptor with pyrin domain containing 3
PBMC: Peripheral blood mononuclear cell
PICs: Pro-inflammatory molecules
pro-caspase-1: Pro-cysteine aspartic acid protease-1
PVN: Hypothalamic paraventricular nucleus
SD rats: Sprague-Dawley rats
TH: Tyrosine hydroxylase
TLR4: Toll-like receptor 4
TNF-α: Tumor necrosis factor-α
VCAM-1: Vascular cell adhesion molecule 1
