# Supplementary material for: Central blockade of NLRP3 reduces blood pressure via regulating inflammation microenvironment and neurohormonal excitation in salt-induced prehypertensive rats
Source: J Neuroinflammation. 2018 Mar 24;15:95. doi: 10.1186/s12974-018-1131-7 (PMC5866519; doi:10.1186/s12974-018-1131-7)
Supplement: Supplementary file 1 — Figure S1. Salt-induced prehypertension is partly due to the role of NLRP3 in PVN, via an inflammatory mechanism. Blockade of brain NLRP3 attenuates prehypertensive response, possibly via downregulating the cascade reaction triggered by inflammation and restoring the balance of neurotransmitters (PPTX 203 kb) [file 12974_2018_1131_MOESM1_ESM.pptx]

## Slide 1
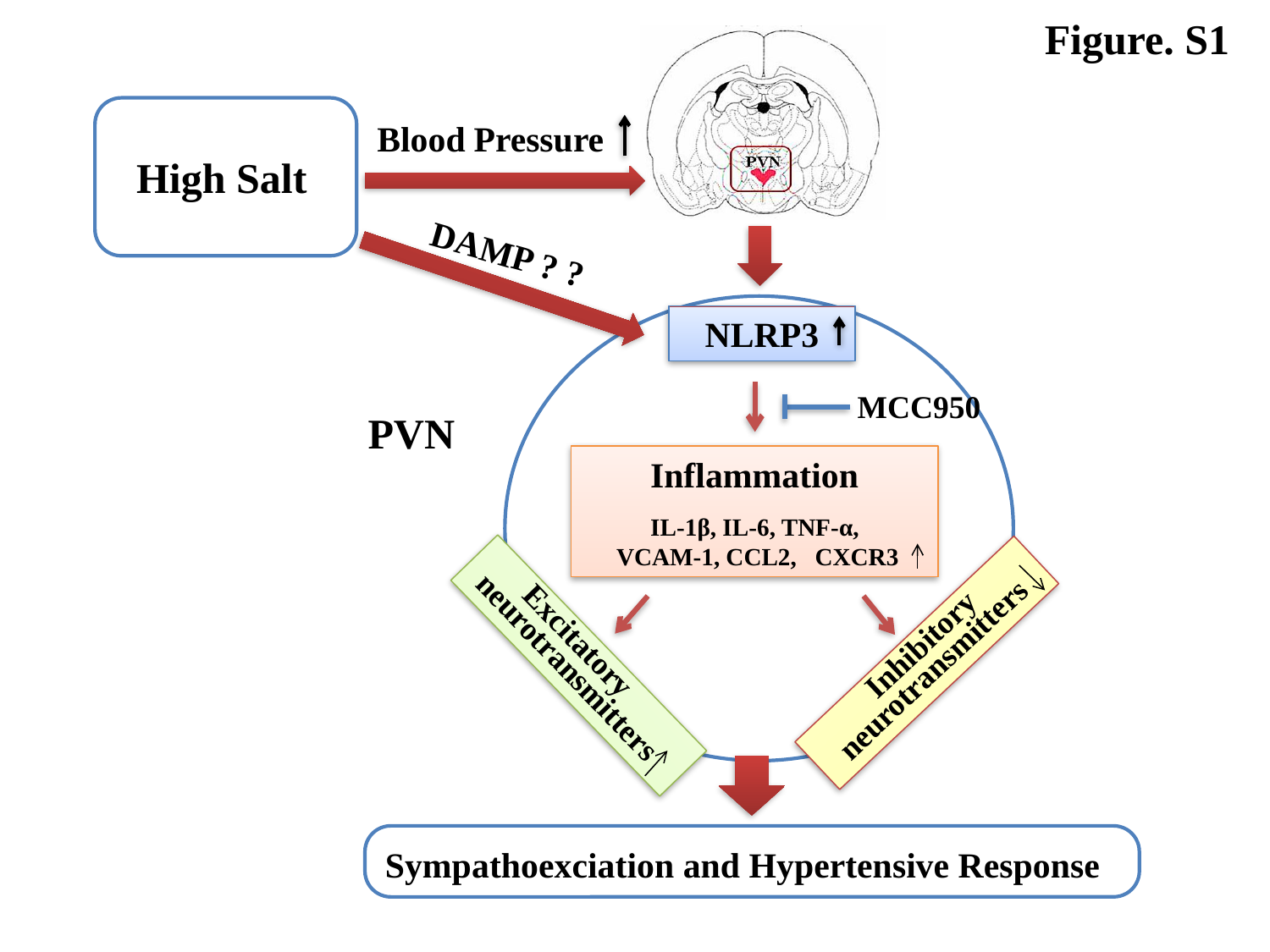

Figure. S1
 High Salt
Blood Pressure
DAMP ? ?
NLRP3
MCC950
 PVN
Inflammation
IL-1β, IL-6, TNF-α,
 VCAM-1, CCL2, CXCR3
 Excitatory
 neurotransmitters
 Inhibitory
neurotransmitters
Sympathoexciation and Hypertensive Response
